# Dr. Geo. W. McElhaney

**Published:** 1893-12

**Authors:** 


DR. GEO. W. MCELHANEY IS DEAD.—In our last issue was briefly mentioned the fact that Dr. McElhaney had been suddenly attacked with paralysis in the right side, but we were unable to obtain further particulars before going to press. We now have to announce the sad news of his untimely death, which occurred at his home, in Columbus, Ga., on the morning of September 15, a few days after the fatal attack. Thus another terrible example of the mysterious will of Providence more forcibly impresses us with the fact that we know not the day or the hour that has been appointed. The last time I saw him he was at Chicago attending to his duties as a member of the Executive Committee of the World's Dental Congress, apparently in the best of health and spirits. Those who saw him there and looked into his kindly beaming face and felt the warm grasp of his hand in friendship will see him no more on earth, for his spirit has taken its flight to that home above, prepared for him as a reward for a life well and nobly spent on earth.

Dr. Geo. W. McElhaney was born at Ellerslie, Harris County, Ga., on August 13, 1843. His father died when he was only seven years of age. At the age of ten years he was taken to Auburn, Ala., where he was reared and educated in the family of Dr. F. G. McElhaney. The war between the States breaking out, he entered the Confederate army at the age of sixteen and went to Virginia.

He was there only a short time when he was stricken with fever and had to be carried home. On regaining his health he again joined the army, enlisting in the cavalry service under General Lee, of Mississippi. He served through the whole war, making a good, faithful soldier.

After the war he entered the office of Dr. F. G. McElhaney to prepare himself for the practice of dentistry. He first engaged in practice at West Point, Ga., and was married a short time after to Miss Katie Moore. Some years after, he graduated and received the degree of D. D. S. from the University of Tennessee, at Nashville. It was in 1881 that Dr. McElhaney moved to Columbus, Ga., and associated himself with Dr. W. F. Tignor in the practice of his profession.

After a dissolution of the firm of Tignor & McElhaney, he opened an office to himself and built up a flourishing practice, which he held to the time of his death. Dr. McElhaney has held many positions of trust and honor, and was a useful citizen in his community.

He has been president of the Georgia State Dental Society and of the Southern Dental Association. He was a prominent member of the American Dental Association, and often held positions of honor in that body. He was also a member of the Dental Protective Association, and for many years served as a member of the Georgia State Board of Dental Examiners.

When the movement was inaugurated to hold a World's Dental Congress, his ability as a businezs man suggested him as suitable to make one of the Executive Committee of that body, which position he filled fully and creditably.

Dr. McElhaney, in addition to all that has been said, was a temperate, moral Christian man, and early associated himself with institutions of that character. He was a prominent member of the Methodist Episcopal Church, He was also an enthusiastic Mason, having been Grand Commander of the Grand Commandery of Knights Templar of Georgia, and also Past Commander of St. Aldemar Commandery, No. 3, of Columbus. Last year he represented the Grand Commandery of the State at the triennial conclave of the order at Denver, Col.

The body of the deceased was buried at Auburn, Ala., shrouded in the uniform of the rank of Past Grand Commander Knights Templar, and on the lid of the casket, almost hidden beneath the beautiful floral tributes, were placed the helmet and sword of the office. The dentists of the city turned out in a body to pay a last tribute to their beloved professional brother, and afterwards met and passed suitable resolutions, which we publish below.

The body was carried to Auburn on a special car under an escort from St. Aldemar Commandery, No. 3, and was laid to rest with the ceremonies of the order.

This State and the profession will feel the loss sustained by the death of Dr. McElhaney, and to the bereaved and sorrowing wife the true sympathy of a vast concourse of friends go out, in this time of grief and sadness. H. H. J.

The following preamble and resolutions were passed by the dentists of Columbus, Ga., on the death of Dr. McElhaney:

"WHEREAS, The grim monster Death has invaded the ranks of our profession and removed from our midst a conspicuous and honored member, Dr. George W. McElhaney, it is appropriate that some public expression should be given of our keen regret at the loss we have sustained, therefore be it

"*Resolved*, That in the death of Dr. George W. McElhaney the dental profession of Columbus has been deprived of a valued and enthusiastic member, a generous hearted and affable friend, and the community of a useful and honorable citizen.

His extraordinary ability and skill as an operator placed him among the foremost dentists of the South. His zeal and love for his profession were recognized in his work in promoting its highest interests, and his unswerving duty as a member of the Georgia State and Southern Dental Societies will ever be remembered by his associates.

"*Resolved*, That our sincerest sympathies are tendered his bereaved wife in her deep affliction.

"*Resolvcd*, That this simple tribute to his worth as a man and his fame as a dentist be published in the daily papers as expressive of his sentiments.

"C. T. OSBORN,
"W. T. POOL,
"AUG. BURGHARD,
"*Committee.*"

—*Southern Dental Journal.*

